# Legitimacy and Geriatrics: Making Space for Geriatric Care in a National Healthcare System

**DOI:** 10.1093/geroni/igaf122.1228

**Published:** 2025-12-31

**Authors:** Jessica Riley, Eileen Dryden, Lauren Moo, Meaghan Kennedy, Camilla Pimentel, William Hung

**Affiliations:** Veterans Health Administration, Quincy, Massachusetts, United States; Veterans Health Administration, Bedford, Massachusetts, United States; VA Bedford Healthcare System, Bedford, Massachusetts, United States; VA Bedford Health Care System, Bedford, Massachusetts, United States; VA Bedford Health Care System, Bedford, Massachusetts, United States; James J Peters VA Medical Center, New York, New York, United States

## Abstract

This presentation uses the lens of medical anthropology to better understand how geriatric services are established and maintained within the Veterans Health Administration (VHA) system. Specifically, we look to Margaret Locks theoretical concept of ‘biomedical legitimacy’ in biomedicine to examine the challenges and strategies involved in implementing and maintaining geriatric programs aimed at serving rural Veterans within a complex healthcare landscape. Particularly we build on Lock’s biomedical legitimacy concept by considering how contextual factors, within a national healthcare system setting, influence the availability of geriatric services. We conducted semi-structured interviews with 13 staff members from four VA Clinical Resource Hubs (including a range of professional backgrounds such as psychiatry, family medicine, and geriatrics) and 11 former national, regional, and facility leaders in the VHA representing over 250 cumulative years of experience. Data were initially analyzed through team-based rapid qualitative analysis and then examined using the construct of “legitimacy.” Some of the findings include facilitators and barriers staff experienced while advocating for geriatric care as part of their region’s service line, some of which included leadership support and availability of clinicians to provide specialized geriatric care. This analysis provides a broader understanding of the systemic and logistical considerations that impact the delivery of geriatric care and highlights potential pathways for strengthening support for aging Veterans within the VA system and beyond.

